# Development of a Structural Equation Model to Examine the Relationships between Genetic Polymorphisms and Cardiovascular Risk Factors

**DOI:** 10.3390/nu15112470

**Published:** 2023-05-25

**Authors:** Joseph Musonda Chalwe, Christa Grobler, Wilna Oldewage-Theron

**Affiliations:** 1 Department of Health Sciences, Vaal University of Technology, Private Bag X021, Vanderbijlpark 1900, South Africa; christa@vut.ac.za; 2 Department of Nutritional Sciences, Texas Tech University (TTU), Lubbock, TX 79409, USA; 3 Department of Sustainable Food Systems & Development, University of the Free State, Bloemfontein 9300, South Africa

**Keywords:** structural equation model, genetic polymorphisms, dyslipidemia, cardiometabolic, cardiovascular risk, elderly, South Africa

## Abstract

Genome-wide association studies (GWASs) have been used to discover genetic polymorphisms that affect cardiovascular diseases (CVDs). Structural equation modelling (SEM) has been identified as a robust multivariate analysis tool. However, there is a paucity of research that has conducted SEM in African populations. The purpose of this study was to create a model that may be used to examine the relationships between genetic polymorphisms and their respective cardiovascular risk (CVR) factors. The procedure involved three steps. Firstly, the creation of latent variables and the hypothesis model. Next, confirmatory factor analysis (CFA) to examine the relationships between the latent variables, SNPs, dyslipidemia and metabolic syndrome, with their respective indicators. Then finally, model fitting using JASP statistical software v.0.16.4.0. The indicators for the SNPs and dyslipidemia all indicated significant factor loadings, −0.96 to 0.91 (*p* = <0.001) and 0.92 to 0.96 (*p* ≤ 0.001), respectively. The indicators for metabolic syndrome also had significant coefficients of 0.20 (*p* = 0.673), 0.36 (*p* = 0.645) and 0.15 (*p* = 0.576), but they were not statistically significant. There were no significant relationships observed between the SNPs, dyslipidemia and metabolic syndrome. The SEM produced an acceptable model according to the fit indices.

## 1. Introduction

Cardiovascular diseases (CVDs) are still the primary cause of premature mortality worldwide [1,2]. For this reason, prevention approaches by identifying individuals with an increased risk is the utmost concern that needs to be addressed [1,3]. Some progress has been made in the development of prediction tools for CVD outcomes. Prediction tools such as the Framingham CVD risk score (FRS) model [4] and the Suita score model for CVDs [5] have been used in many studies. Despite their success in the prediction of CVD risks, they have been shown to be inconvenient in primary care, as medical professionals need to calculate the risks of each component separately then incorporate all the factors. Additionally, they have been reported to overestimate the CVD risks in some cases [4,5].

Various factors have been reported to contribute to the development of CVDs. Evidence has shown that some of these factors are unique to an individual. Genetics has been considered as a factor that impacts the pathogenesis of CVDs because mutations alter the structure and quantity of the gene products, ultimately affecting the function of the gene products such as cardiovascular risk factors (CVR) [6]. For example, a report by Hongmei and co-authors [7] demonstrated that approximately 50 risk points in the human genome can affect the incidence of CVD. Furthermore, large-scale GWASs have shown that some single-nucleotide polymorphisms (SNPs) not only increase the risk of CVD but also seem to have pleiotropic effects. Pleiotropic means demonstrating a strong association with other human diseases [8]. The extent to which genetics affect an individual’s health, however, depends on factors such as age, inception and disease type [9].

At molecular a level, GWASs have also been effectively applied to discover genetic polymorphisms that affect CVDs. The limitations of some of the techniques in GWASs are that only important SNPs that are related to specific diseases are reported, and there are concerns that these techniques might ultimately link the entire genome to disease susceptibilities [10]. For this reason, the analyses of the relationships between these genotypes and the CVD outcomes are still unclear because of the complexity of relationship between genetics and environmental factors that are yet to be explored [11,12]. SEM tools have, however, been determined to have more statistical power because of the categorized modelling functionality that includes both latent and continuous variables [13].

SEM has been successfully used in a variety of fields such as psychiatry research [14] and genetic analysis studies [15]. Most of these studies have, nevertheless, only been conducted in western [16] or Asian countries [17]. Furthermore, many studies did not analyze multiple SNPs and their individual or combined roles in these diseases simultaneously. There is a paucity in the number of studies that have conducted SEM in African populations [18]. CVDs have become a burden in African countries including peri-urban and rural areas because of the socio-economic transition and adoption of western diets [19]. Therefore, we aimed to create a model that may be used to examine the relationship between genetic polymorphisms and cardiovascular risk (CVR) factors.

## 2. Materials and Methods

### 2.1. Study Design and Sample

This study used a convenience sampling method of the elderly (n = 61) who attend a day care in a black peri-urban population of Sharpeville, Vaal region, South Africa. Fasting blood samples were collected on the same day (within 2 h) from the elderly participants who met the inclusion criteria of age 60 years or older and voluntary attendees of the Sharpeville day care centre, as well as signing the informed consent form for participation. Participants who were not able to provide substantial information to complete the consent process due to conditions such as dementia were excluded from this study.

### 2.2. DNA Extraction and Genotyping

Genomic DNA was extracted from peripheral blood samples using the Quick-DNA™ Miniprep DNA purification kit (Zymo Research, Irvine, CA, USA) following the manufacturer’s instructions. Microtubes were placed onto a rack using a cool block. A total of 200 μL of each sample was added to a 1.5 mL microcentrifuge tube. Next, 200 μL of BioFluid & Cell Buffer (Red) and 20 μL of Proteinase K was then added to the same 1.5 mL microcentrifuge tube. The contents were mixed using a vortex for 15 s and then incubated at 55 °C for 10 min. One volume of the genomic binding buffer was added to the digested sample. The mixture was then mixed for another 15 s. The mixture was transferred to a Zymo-Spin™ IIC-XL Column in a collection tube. This was then followed by centrifugation at ≥12,000× *g* for 1 min. The collection tube with the flow through was discarded. A total of 400 μL of the DNA pre-wash buffer was added to the same spin column in a new collection tube and centrifuged at ≥12,000× *g* for 1 min. The collection tube was then emptied. The mixture was washed in two steps by adding 700 μL and 200 μL g-DNA wash buffers to the spin column, respectively, with subsequent centrifugation at ≥12,000× *g* for 1 min. The collection tube was then emptied again after each wash. The spin column was transferred to a clean microcentrifuge tube and 50 μL DNA elution buffer was added directly on the matrix. This was followed by incubation for 5 min at room temperature, then centrifugation at maximum speed for 1 min to elute the DNA. The DNA concentration was measured by spectrophotometry using the NanoDrop^®^ 2000 (NanoDrop Technologies, Wilmington, DE, USA) and DNA purity was determined by 1% agarose gel analysis using the A260/A280 ratio. A ratio of ~1.8 is generally accepted as “pure” for DNA.

Genotyping was outsourced to Inqaba Biotechnical Industries (Pty) Ltd., Pretoria, South Africa, which uses the Agena Bioscience, MassARRAY genotyping system, based on single base-extension or cleavage chemistry in conjunction with matrix-assisted laser desorption/ionization–time of flight (MALDI–TOF) mass spectrometry. Current advances in GWASs have enabled the identification of several SNPs that have been linked to human diseases including CVDs [10]. The SNPs for this study were identified by conducting a literature search of PubMed databases and relevant articles to determine the specific SNPs that have an association or influence on the risk factors of CVDs. Following the search, the rs675 of apolipoprotein A-IV (ApoA-IV), rs699 of angiotensinogen (AGT), rs247616 and rs1968905 of cholesteryl ester transfer protein (CETP), rs1801278 of insulin receptor sub-strate-1 (IRS-1), rs1805087 of methylenetetrahydrofolate reductase (MTHFR), rs28362286 and rs67608943 of proprotein convertase subtilisin/kexin type 9 (PCSK9) were selected based on their clinical significance (CVR) and prevalence. These SNPs were therefore analyzed in each sample. They were genotyped using the TaqMan^®^Pre-designed SNP Genotyping Assay Kit (Thermo Fisher Scientific, Foster City, CA, USA). The 20 µL reaction mix consisted of: 1 µL template DNA (15 ng/µL), 10 µL TaqMan^®^Genotyping Master Mix (Cat. # 4371355), 1 µL probe (TaqMan^®^Pre-designed SNP Genotyping Assay), and 8 µL deionized water. The probe was diluted in Tris EDTA buffer (10 mM Tris–HCl (pH 8.0), 0.1 mM EDTA) (1:1) before the reaction. Polymerase Chain Reaction (PCR) was performed according to the manufacturer’s specifications using the Bio-Rad CFX Real-Time PCR System (Hercules, CA, USA). The primers and probes were purchased form Inqaba Biotech (Pretoria, South Africa) at concentrations of 10 μM. PCR amplification of the target loci involved amplifying each specific fragment of genomic DNA, which was then genotyped on the Agena MassARRAY (San Diago, CA, USA) platform. A Multiplex PCR cocktail was then prepared according to the manufacturer’s instructions. The products were then measured using the MassARRAY Compact mass spectrometer and Agena real-time detection software (San Diago, CA, USA). A no-template control was included in every PCR reaction to detect false positive reactions. Detection of rs675, rs699, rs247616, rs1968905, rs1801278, rs1805087, rs28362286 and rs67608943 was successful in the participants. Genotypes of the SNPs were also detected.

### 2.3. Blood Pressure Measurements

Blood pressure (BP) measurements were conducted using a previously reported method [20]. The participants were requested to sit quietly for a minimum of 5 min in a chair with a back support, feet on the floor and arm supported at heart level before the measurements. The Tensoval^®^ duo control monitor was wrapped firmly around the right arm wrist consistently to measure systolic and diastolic BP readings in duplicate on two different days at the same time by a Health Professions Council of South Africa (HPCSA)-registered nurse. For accuracy, the mean systolic and mean diastolic readings were then determined and recorded. Hypertension was specified according to the South African Hypertension Society (SAHS) guidelines at ≥140/90 mmHg.

### 2.4. Biochemical Measurements

Serum was prepared from the vacutainer blood collection tubes by centrifugation of whole blood for 15 min at 4500 rpm. Aliquots of the serum were then stored in the laboratory at −20 °C until analysis. Serum apolipoprotein A-IV (Apo A-IV), apolipoprotein B (ApoB), high-density lipoprotein cholesterol (HDL-C), homocysteine (hcy), insulin, lipoprotein (a) (Lipo (a)), low-density lipoprotein cholesterol (LDL-C), total cholesterol (TC) and triglyceride (TG) were measured quantitatively using standardized commercial kits (Thermo Fisher Scientific, USA) on a Konelab 20i Thermo Scientific autoanalyzer (Thermo Fisher Scientific, USA). The Konelab 20i Thermo Scientific autoanalyzer is a clinical chemistry analyzer that works on colorimetric and immunoturbidimetric principles. Lyophilised calibrator and quality control samples from the manufacturer were reconstituted before use and run before the serum samples to validate the tests. Proprotein convertase subtilisin/kexin type 9 (PCSK9) was assessed using an internationally standardized kit from EIAab^®^ (Eiaab, Wuhan, China). The kit consisted of assay plates with micro-titre wells pre-coated with antibodies specific to PCSK9 (Eiaab, Wuhan, China). Reagent preparation included reconstitution of a wash buffer with distilled water, reconstituting a standard with sample diluent, and preparation of a detection reagent A and B working solution with their corresponding assay diluents (Eiaab, Wuhan, China). Washing steps were conducted with the W206—Microplate Washer (Chengdu Empsun Medical Technology Co., Ltd., Chengdu, China). The final absorbance readings were conducted using a microplate reader (Rayto, RT-2100C, Shenzhen, China). The plate was immediately read using the M201—ELISA microplate reader (Chengdu Empsun Medical Technology Co., Ltd., Sichuan, China). These calibration standards were run in duplicate from which a standard curve was generated to determine serum PCSK9 levels.

### 2.5. Development of Latent Variables

In this analysis, the SNP names rs1801278 (SP1), rs1805087 (SP2), rs1968905 (SP3), rs247616 (SP4), rs28362286 (SP5), rs675 (SP6) and rs699 (SP7) were denoted to enable shorter identification in the JASP statistical software v.0.16.4.0 for Windows (JASP Team, 2023). The preliminary analysis using a covariance matrix assay (exploratory factor analysis) showed that in our population only SP1 (IRS1), SP2 (MTHFR), SP3 (CETP), SP4 (CETP) and SP5 (PCSK9) were strongly correlated; for this reason, only these five were used as indicators for SNPs. Initially, we attempted to use the usual CVD risk indicators for an extended lipid profile (elevated TC, TG, LDL-C, HDL-C, Apo A-I, ApoB and Lipo (a)) [21] and metabolic syndrome (obesity, elevated glucose, BP and lipidaemia) [22]. Nevertheless, we found that only TC, LDL-C, ApoB and Lipo (a) had statistically significant loadings in the model; therefore, these four were used as indicators for dyslipidemia. Furthermore, only glucose, BP and PCSK9 had statistically significant loadings in the model; therefore, these three were used as indicators for metabolic syndrome. Hence, the other indicators were excluded from the final diagram.

### 2.6. Data Analysis and Validation (Structural Equation Modeling)

**Figure 1:**
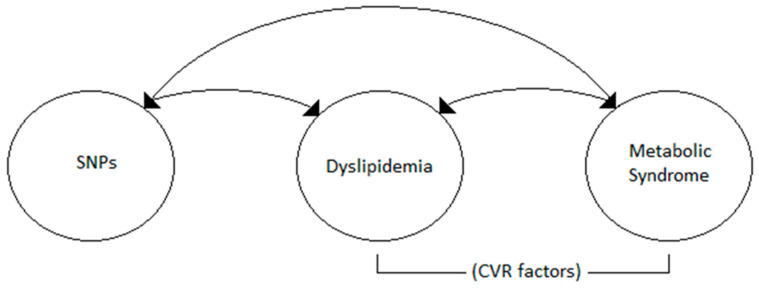
Hypothesized model.

All the raw data was cleaned and captured in Microsoft Office Excel (Microsoft Corp, Redmon, WA, USA). A conceptual framework was developed to indicate the possible relationships between the latent variables (Figure 1). SEM was performed in our sample based on the hypothesized model in Figure 1. The directional arrows (single-headed) imply regression, while the double-headed curved arrows in the figure imply a covariance between two variables (correlation) [23].

The procedure involved three steps. Firstly, we created the latent variables and the hypothesis model. The variables were derived from the SNPs and CVR factors in our population. The SNPs for this study were selected based on their association to CVR in the literature; namely, rs675 (ApoA-IV), rs699 (AGT), rs247616 and rs1968905 (CETP), rs1801278 (IRS-1), rs1805087 (MTHFR), rs28362286 and rs67608943 (PCSK9). The CVR factors that were investigated are glucose, blood pressure (BP), apolipoprotein A-1 (ApoA1), apolipoprotein B (ApoB), total cholesterol (TC), high-density lipoprotein cholesterol (HDL-C), insulin, lipoprotein (a) (Lipo(a)), low-density lipoprotein cholesterol (LDL-C), homocysteine (hcy), proprotein convertase subtilisin/kexin type 9 (PCSK9) and triglyceride (TG). This was then followed by confirmatory factor analysis (CFA) which was used to examine the relationship between the latent variables: SNPs, dyslipidemia and metabolic syndrome and their respective indicators (CVR). Once this was performed, the model was then fit using the JASP statistical software v.0.16.4.0 for Windows (JASP Team, 2023) using the lavaan (v. 0.6–1) package for R [24]. 

Following the SEM, we estimated and validated the model using the chi-square test (χ2), root mean square error of approximation (RMSEA), the standardized root mean square residual (SRMR), comparative fit index (CFI), goodness-of-fit index (GFI) and Tucker–Lewis index (TLI). The chi-square test (χ2) measures a relationship between two categorical variables. Hence, a non-significant difference is ideal [25,26,27]. The root mean square error of approximation (RMSEA) measures the projected difference between the population and model-implied population covariance matrices per degree of freedom. A value of zero signifies a perfect fit while higher values indicate the lack of fit [26,28,29]. The standardized root mean square residual (SRMR) measures the difference between the observed correlation and the correlation matrix. The comparative fit index (CFI) explores the model fit by assessing the difference between the data and the hypothesized model. It ranges from 0.0 to 1.0. A higher CFI value implies a better model fit [22]. The goodness-of-fit index (GFI) evaluates the difference between the sample covariance matrix (S) and the estimated covariance. It also ranges from 0.0 to 1.0 [30,31]. The Tucker–Lewis index (TLI) is a gradational fit index that is generally used in linear mean and covariance structure modeling. It also ranges from 0.0 to 1.0 [25,26,32]. The term “factor loadings” indicates the standard coefficients that are observed between the variables and the latent variables [24,33,34]. We hypothesized relationships between the genetic polymorphisms, dyslipidemia and metabolic syndrome as risk factors for CVD based on the literature.

## 3. Results

**Figure 2:**
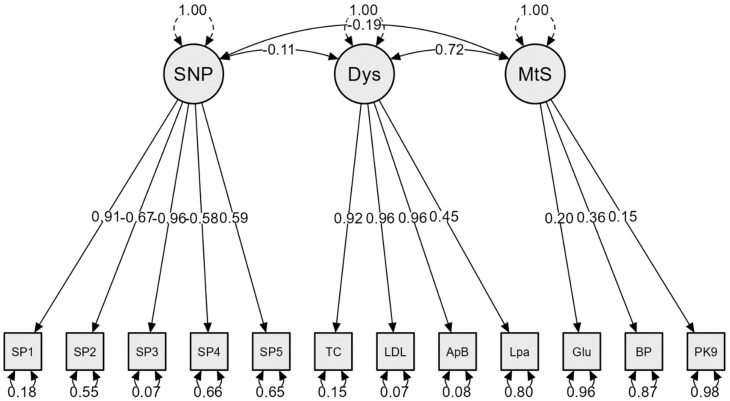
Structural equation model of the relationships between the SNPs and CVR factors (dyslipidemia and metabolic syndrome). **KEY:** Single-nucleotide polymorphism (SNP); SP1—rs1801278; SP2—rs1805087; SP3—rs247616; SP4—rs1968905; SP5—rs28362286; dyslipidemia (Dys); glucose (glu); metabolic syndrome (MtS); blood pressure (BP); apolipoprotein B (ApB); total cholesterol (TC); lipoprotein (a) (Lpa), low-density lipoprotein cholesterol (LDL); proprotein convertase subtilisin/kexin type 9 (PK9).

The factor loadings and standardized coefficients as shown in Figure 2 and Table 1 provide information on the interactions between the latent variables and their respective indicators. As expected, the indicators for the SNPs and dyslipidemia all indicated significant factor loadings, with standardized coefficients ranging from −0.96 to 0.91 (*p* = <0.001) and 0.92 to 0.96 (*p* ≤ 0.001), respectively. The indicators for metabolic syndrome also had significant coefficients of 0.20 (*p* = 0.673), 0.36 (*p* = 0.645) and 0.15 (*p* = 0.576), but they were not statistically significant.

There were no significant relationships observed between the SNPs (based on rs1801278, rs1805087, rs247616, rs1968905 and rs28362286), dyslipidemia (based on TC, LDL-C, ApoB and Lipo (a)) and metabolic syndrome (based on glucose, BP and PCSK9). The findings of the SEM analysis that explored the relationships between the SNPs, dyslipidemia and metabolic syndrome are shown in Table 2, and the goodness-of-fit of the model is summarized in Table 3. 

### Goodness-of-Fit of the Model

The chi-square statistic for the model was 118.483 with 51 degrees of freedom (*p* ≤ 0.001). Regarding the goodness-of-fit indices, RMSEA was 0.164 (90% CI: 0.126–0.203) suggesting a marginal fit. This is because we had a small population and RMSEA has been reported to be affected by sample size [26,28,29,35]. The SRMR and CFI were 0.087 and 0.825, respectively, both suggesting an acceptable fit. However, the TLI was 0.773 suggesting a mediocre fit. The SEM analysis produced an overall acceptable model according to the fit indices [36].

## 4. Discussion

The goal of our study was to develop and explore the relationships between genetic polymorphisms and cardiovascular risk factors in an elderly population from a peri-urban community. Studies have reported that rs1801278 [37], rs1805087 [38], rs247616 [39] and rs28362286 [40] have demonstrated a clinical significance in the development of CVDs. In this study, these variables were used as a collective to measure SNPs, and they were statistically significant. This indicated that they were good measures of SNPs. The indicators for dyslipidemia in our study were elevated serum TC, LDL-C, ApoB and Lipo (a) which are some known risk factors for CVD [41]. As anticipated, these parameters had high loadings for dyslipidemia (*p* = 0.001). Several studies have shown a high prevalence of dyslipidemia in South Africa [42,43,44]. For example, in a multinational study, Dave and co-authors [42] reported that South Africa had the highest prevalence of dyslipidemia (89.9%) compared to other countries such as Kenya, Mozambique, Zambia, Senegal, Uganda, Togo and Malawi. Dyslipidemia is a known major risk factor for CVD with adverse outcomes and should, therefore, be controlled in the elderly population. Literature has shown that glucose, BP and PCSK9 are some parameters that may be used to describe metabolic syndrome [45]. In our study, these were combined to create a measure for metabolic syndrome. It was surprising to see that even though these variables had loadings for metabolic syndrome, they were not statistically significant. Previous studies [46,47,48] have shown a high prevalence of metabolic syndrome in South Africa which increases with age. This presents an increased risk for CVDs, especially in the elderly. 

According to our hypothesized model, we expected to see an effect of the SNPs on dyslipidemia [39,49] and metabolic syndrome [37,38,40,50] based on literature. Surprisingly, in our model, no significant relationship was observed between the SNPs, dyslipidemia and metabolic syndrome and eventually CVR. This finding affirms our assumption in the previous chapter that the heterozygous and homozygous genotypes of the SNPs that we investigated may have independent and possible collective roles in increasing the risk of CVDs. Our findings lend support to a report by Paththinige and co-authors [51] who stated that a majority of the genetic polymorphisms that have been discovered have a small effect on the outcome of CVR-linked conditions such as dyslipidemia and metabolic syndrome. 

There are, however, some conflicting results on the effect of genetic polymorphisms on the different markers of CVDs. This might be the other reason why there is inadequate information on the mechanisms and inheritance of these complex conditions. For example, there is a body of evidence that suggests that some genetic mutations or polymorphisms that occur in the genes of the lipid profile parameters [51,52,53] and metabolic markers [54,55,56,57] have a minimal or no effect on the outcomes of these CVR factors. While on the other hand, some studies have demonstrated a significant effect of polymorphisms on the CVR factors such as the lipid profile [58,59] and metabolic markers [60,61]. This inconsistency in all these reports is what makes the exact role of these polymorphisms on CVR factors still unclear and, therefore, warrants further investigations using a standardized approach with more robust genetic tools such as SEM [13]. 

CVDs are a growing challenge with devastating outcomes. Studies have been conducted to try and understand these conditions but there is an urgent need to further investigate them at a molecular genetic level [2,62]. The discovery and development of new approaches to study the molecular mechanisms and pathways will offer new understandings into these diseases. This may facilitate the production of new prevention and treatment approaches. Additionally, this might give insights into the discovery of genetic markers or models that may be used for screening and monitoring CVDs [62]. Genome-wide association studies (GWASs) and nutrigenomics have been effective in producing a vast amount of data from genes, polymorphisms and their interactions in diseases such as type 2 diabetes, hypertension and CVDs [63,64]. SEM has been identified as a robust multivariate analysis tool that is demonstrated to have an excessive number of possibilities in research while we see a rise in the amount of genomic data globally [13].

We propose a model that may provide an opportunity to better understand the complex interaction of genetic polymorphisms in the CETP, IRS1, MTHFR and PCSK9 genes with the ApoB, BP, glucose, TC, LDL-C, Lipo (a) and PCSK9 levels in a black elderly population from Sharpeville in South Africa. Models such as this may enable early detection of CVDs, especially in asymptomatic individuals, allow precise treatment and lessen the mortality efforts by the department of health or policy makers [51]. 

From published reports, it appears that the use of indices to assess the fitness of a model is still adaptable. While there is no benchmark, it is evident that the more fit indices are applied to a model, the better for its acceptance. Hu and Bentler [26] recommend that at least two fit indices should be used in combination. In our study, we reported five indices; namely, CFI, TLI, RMSEA, SRMR and GFI. Table 1 shows all the values which are in line with the combined fit indices, suggesting that our proposed model is acceptable. We also presume that the fit index of our model would have been even further improved with a bigger sample size, better dispersion of the variables (polymorphisms and CVR factors) and a lesser amount of missing data as shown in the literature [65,66,67,68]. To date, there are several recommended cutoff values for various indices, but none serve as the determining criteria for all applications [26,29,33,36,65,69]. The resulting model had acceptable goodness-of-fit measures.

## 5. Conclusions

The novelty of the present study is that we developed a model to explain some of the mechanisms that link genetic polymorphisms with the risk factors of CVD (dyslipidemia and metabolic syndrome). Prior to this study, there were no reports that measured the pathways that relate the variables of dyslipidemia and metabolic syndrome in this elderly population. This study used SEM which enabled simultaneous analysis of a combination of different SNPs from different genes with an extended number of different CVR factors. Additionally, our findings supplement insights into an area with a paucity of information known as nutrigenomics. We recommend further studies with such models in diverse populations with larger samples to clarify and directly highlight the exact roles of genetic polymorphisms on the CVR factors. Additionally, we recommend potential interventions by the ministry of health and policy makers such as awareness of dyslipidemia and metabolic syndrome, regular screening of such conditions, promotion of physical activity and facilitation of access to treatment.

## Figures and Tables

**Table 1:** Relationship between latent variables and their indicators.

Latent Variable	Indicator	Factor Loading	*p*-Value (Significance)
SNPs	SP1	0.91	<0.001
	SP2	−0.67	<0.001
	SP3	−0.96	<0.001
	SP4	−0.58	<0.001
	SP5	0.59	<0.001
Dyslipidemia	TC	0.92	<0.001
	LDL-C	0.96	<0.001
	ApoB	0.96	<0.001
	Lipo (a)	0.45	0.001
Metabolic syndrome	Glucose	0.20	0.673
	BP	0.36	0.645
	PCSK9	0.15	0.576

**KEY:** SP1—rs1801278; SP2—rs1805087; SP3—rs247616; SP4—rs1968905; SP5—rs28362286. Statistical significance = *p* < 0.05; single-nucleotide polymorphism (SNP); blood pressure (BP); apolipoprotein B (ApoB); total cholesterol (TC); lipoprotein (a) (Lipo(a)), low-density lipoprotein cholesterol (LDL-C); proprotein convertase subtilisin/kexin type 9 (PCSK9).

**Table 2:** SEM of the relationships between the SNPs and the CVR factors.

Pathway	Association	*p*-Value (Significance)
SNPs ↔ Dys	−0.114	0.440
SNPs ↔ MetS	−0.194	0.612
Dys ↔ MetS	0.719	0.619

Statistical significance = *p* < 0.05; single-nucleotide polymorphism (SNP); dyslipidemia (Dys); metabolic syndrome (MetS).

**Table 3:** Model fit.

Model	x^2^	df	RMSEA	RMSEA 90% CI	SRMR	CFI	TLI
SEM	118.483	51	0.164	0.126–0.203	0.087	0.825	0.773
	(*p* ≤ 0.001)		Marginal		Acceptable	Good	Moderate/OK

## Data Availability

The data that supports the results of this study are available from the corresponding author, J.M.C., upon request.
